# C-reactive protein as an early predictor of COVID-19 severity

**DOI:** 10.5937/jomb0-27554

**Published:** 2020-10-02

**Authors:** Maryame Ahnach, Saad Zbiri, Sara Nejjari, Fadwa Ousti, Chafik Elkettani

**Affiliations:** 1 Mohammed VI University of Health Sciences (UM6SS), Cheikh Khalifa International University Hospital, Department of Hematology, Casablanca, Morocco; 2 Mohammed VI University of Health Sciences (UM6SS), International School of Public Health, Laboratory of Medical Evaluation and Health Economics, Casablanca, Morocco; 3 Mohammed VI University of Health Sciences (UM6SS), National Reference Laboratory, Casablanca, Morocco; 4 Mohammed VI University of Health Sciences (UM6SS), Cheikh Khalifa International University Hospital, Department of Anesthesiology and Reanimation, Casablanca, Morocco

**Keywords:** COVID-2019, SARS-CoV-2, C-reactive protein, early predictor, severity, ozbiljnost, rani pokazatelj, C-reaktivni protein, SARS-CoV-2, COVID-2019

## Abstract

**Background:**

Data for predicting severity of patients with COVID-19 infection are sparse and still under investigation. We retrospectively studied whether the admission serum C-reactive protein level (CRP) can serve as nearly predictor of disease severity during COVID-19 infection in comparison with other hematologic and inflammatory markers.

**Methods:**

We included all consecutive patients who were admitted in Cheikh Khalifa International University Hospital, Casablanca, Morocco, between February to April 2020, with a confirmed diagnosis of COVID-19 infection using SARS-CoV-2 viral nucleic acid via RT-PCR. The complete blood count and serum CRP level were routinely measured on admission. All clinical and laboratory data of patients were collected and analyzed. The classification of the disease severity was in accordance with the clinical classification of the WHO interim guidance, and the management of patients were adapted to the national management guideline. We estimated receiver operating characteristic (ROC) curves of blood routine parameters as well as their association with COVID-19 disease severity.

**Results:**

145 COVID-19 patients were included in the study. The median age (range) was 50 (32-63) years, and 75 (51.7%) were men. 101 patients were classified in the non-severe group and 44 patients in the severe group. Based on disease severity, significant differences were observed in the age, gender, comorbidities, and respiratory symptom. Similarly, the biological analysis found significant differences for the neutrophil count, lymphocyte count, eosinophil count, and CRP level. However, according to ROC curves of these laboratory biomarkers, the AUC of CRP at 0.872 was significantly higher than all other parameters. Further, CRP was independently associated with severity of COVID-19 disease (OR = 1.11, 95% IC (1.01-1.22) and or = 1.13, 95% IC (1.04-1.23)).

**Conclusions:**

This study found that the CRP level at admission represent a simple and independent factor that can be useful for early detection of severity during COVID-19 and the easy guidance of primary care.

## Abstract (sr)

**Uvod:**

Podaci za predviđanje težine stanja pacijenata sa infekcijom COVID-19 su retki i još uvek se istražuju. Retrospektivno smo istražili da li nivo C-reaktivnog proteina (CRP) može da posluži kao rani indikator ozbiljnosti bolesti pri infekciji virusom COVID-19 u poređenju sa drugim hematološkim i upalnim markerima.

**Metode:**

Uključili smo sve pacijente koji su uzastopno primljeni u Međunarodnu univerzitetsku bolnicu Šeik Kalifa u Kazablanki, Maroko, u periodu od februara do aprila 2020. godine, sa dijagnozom COVID-19 infekcije potvrđenom pomoću virusne nukleinske kiseline COVID-19 putem RT-PCR. Kompletna krvna slika i nivo seruma CRP rutinski su mereni na prijemu. Svi klinički i laboratorijski podaci pacijenata su prikupljeni i analizirani. Klasifikacija težine bolesti bila je u skladu sa kliničkom klasifikacijom privremenih uputstava SZO, a lečenje pacijenata prilagođeno međunarodnim smernicama. Izvršili smo procenu ROC krive parametara analize krvi kao i njihovu povezanost sa težinom bolesti COVID-19.

**Rezultati:**

U istraživanje je uključeno 145 pacijenata sa infekcijom COVID-19. Srednja vrednost starosti bila je 50 godina (32-63 godine), a 75 pacijenata (51,7%) bili su muškarci. U lakšu grupu svrstan je 101 pacijent, a u težu 44. Na osnovu težine bolesti primećene su značajne razlike u starosti, polu, komorbiditetima i respiratornim simptomima. Slično tome, biološka analiza otkrila je značajne razlike u broju neutrofila, broju limfocita, broju eozinofila i nivou CRP-a. Međutim, prema ROC krivama ovih laboratorijskih biomarkera, AUC (oblast ispod krive) CRP-a na 0,872 bio je značajno veći od svih ostalih parametara. Dalje, CRP je nezavisno povezan sa težinom bolesti COVID-19 (OR = 1,11, 95% IC (1,01-1,22) i or = 1,13, 95% IC (1,04-1,23)).

**Zaključak:**

Ovo istraživanje je otkrilo da nivo CRP-a pri prijemu predstavlja jednostavan i nezavisan faktor koji može biti koristan za rano otkrivanje težine bolesti COVID-19 i lako sprovođenje primarne nege.

## Introduction

The novel coronavirus (COVID-19) pandemic is the defining global health crisis of the moment and the greatest threat we have faced during this century. As a highly contagious virus, the infection has emerged in China in January 2020 [1]. After Asia, it has then rapidly spread globally. The United States, Brazil, Russia, and Europe are the most affected regions today. According to the World Health Organizations'(WHO) data, in May 2020, five million of the global population has been infected, with more than 340,000 deaths [2].

According to the WHO interim guidance [3], the clinical manifestations of COVID-19 disease are heterogeneous, including severe and non-severe forms. The management of patients is therefore adapted to the severity of the clinical situation. According to recent experiences, the majority of infected persons are not severely affected and can recover without medical intervention, whereas a small number of cases need to be carefully treated and hospitalized in an intensive unit [4] [5]. Many publications have documented the clinical, biological, and radiological characteristics of COVID-19 infection, and several international learned societies have developed protocols for disease management, and proposed some prognosis indicators. The biological analysis, especially the inflammatory and hematologic one, represents a major tool in the diagnosis [6], and in the detection of severe forms [7]. Many prognosis factors including lymphocyte count, lactate dehydrogenase, interleukin 6, procalcitonin, and CRP, were evaluated [8], but the predictive power of each of these indicators in disease classification and prognosis remains largely unclear.

Previous studies have indicated that the aberrant host immune response and cytokine storm may play an important role in the severity of COVID-19 [9]. CRP is an acute-phase protein that serves as an early marker of inflammation or infection. The CRP serum level is routinely measured in early diagnosis of pneumonia [10], and some Chinese publications have reported the prognosis value of CRP [11].

In this study, we retrospectively analyzed the clinical and biological characteristics of the COVID-19 infected patients, and investigated the ability of CRP to predict at an earlier time the disease severity, in comparison with other biomarkers.

## Materials and Methods

### Study population and design

We performed a retrospective study including all patients with COVID-19, admitted in the International University Cheikh Khalifa Hospital, during the period from February to April 2020. We included only the laboratory-confirmed cases, as the diagnosis was performed by a real-time reverse-transcriptase polymerase-chain reaction (RT-PCR) assay to test nasal and pharyngeal swab specimens according to the WHO guidance. Epidemiological characteristics including demographics, recent exposure history, clinical symptoms and signs, and laboratory findings, were obtained from electronic medical records (DXcare and LIMS informatics system). We classified the disease severity according to the clinical classification of the WHO interim guidance [3]. Non-severe patients were all patients in mild, moderate, or asymptomatic group, and severe patients were those in a critical or severe group [12] [13]. All patients were managed according to the Moroccan protocol of the Ministry of Health [14].

### Clinical and laboratory data

In terms of epidemiological information, we con sidered patient demographic characteristics including age and gender; comorbidities including hypertension, diabetes, cardiovascular disease, respiratory disease, and other disease; clinical symptoms including fever, general symptom, respiratory symptom, ear, nose and throat (ENT) symptom, and digestive symptom; and clinical outcomes including disease severity and death.

Nasal-pharyngeal swabs and venous blood samples were collected and examined by the National Reference Laboratory, Mohammed VI University of Health Sciences, Casablanca, Morocco. Laboratory confirmation of SARS-CoV-2 was achieved by the RT-PCR assay conducted in accordance with the protocol established by the WHO. Laboratory tests on admission comprised complete blood count, blood chemistry, and biomarkers including leucocyte, neutrophil, lymphocyte, monocyte, eosinophil, hemoglobin, platelet, and CRP.

### Statistical analysis

For the descriptive analysis, we described continuous variables as medians with interquartile ranges (IQRs) and categorical variables as percentages and frequencies. Patients from severe and non-severe risk categories were compared in terms of demographics characteristics, comorbidities, clinical symptoms, and laboratory findings, using Mann-Whitney-Wilcoxon test for continuous variables and using the Fisher exact test for categorical variables.

To determine and compare the accuracy of hematological factors and CRP level on admission in severity prediction, the receiver operating characteristic (ROC) curve analysis was performed, and the difference in the area under the curve (AUC) was tested.

We finally examined the association between CRP level and severity of COVID-19 disease. First, univariate analysis was performed for all variables. Second, multivariate logistic regression was implemented to examine the independent association of CRP level with severity of COVID-19 disease. All significant variables in the univariate analysis were included in the multivariate logistic regression. We also performed stepwise multivariate analysis based on a bidirectional elimination in order to take into account the higher correlation that may exist between some variables particularly those of comorbidities and of laboratory markers. Results were reported as odds ratios (ORs) and 95% confidence intervals (CIs).

All statistical analyses were performed using STATA. All P-values were two-sided, and those < 0.05 were considered as statistically significant.

### Ethics

The study was approved by the institutional ethics board of Cheikh Khalifa IbnZaid International University Hospital. No patient consent was required as the study did include only unidentified information, in accordance with the national law.

## Results

From February to April 2020, 145 COVID-19 patients were admitted to Cheikh Khalifa International University Hospital. Based on the severity of disease, 101 patients were classified in the nonsevere group, and 44 patients were in the severe group. Among the severe cases, 14 patients died.

The characteristics of our population are summarized in Table 1. The median age (range) was 50 (32-63) years, and 75 (51.72%) were men. Of the 145 patients, the most common comorbidities were hypertension (24.83%), other disease (21.38%), diabetes (12.41%), and cardiovascular disease (11.03%). The most common symptoms were respiratory symptom (56.55%), fever (42.76%), fatigue and general symptom (38.62%). Laboratory findings for all patients on admission showed that the median white blood cell count, lymphocyte count, hemoglobin, and CRP level were all in the normal range.

**Table 1:** Characteristics of the study population. ENT, ear, nose and throat; CRP, C-reactive protein.

	Median (IQR) or N (%)
Demographics	
Age, years	50 (32–63)
Male	75 (51.72)
Comorbidities	
Hypertension	36 (24.83)
Diabetes	18 (12.41)
Cardiovascular disease	16 (11.03)
Respiratory disease	14 (9.66)
Other disease	31 (21.38)
Clinical symptoms	
Fever	62 (42.76)
General symptom	56 (38.62)
Respiratory symptom	82 (56.55)
ENT symptom	42 (28.97)
Digestive symptom	34 (23.45)
Blood routine	
Leucocyte (×10^9^ per L)	6.31 (4.88–7.35)
Neutrophil (×10^9^ per L)	3.87 (2.67–5.23)
Lymphocyte (×10^9^ per L)	1.57 (1.02–2.14)
Monocyte (×10^9^ per L)	0.46 (0.36–0.64)
Eosinophil (×10^9^ per L)	0.03 (0.01–0.06)
Haemoglobin (g/L)	139 (129–149)
Platelet (×10^9^ per L)	226 (187–280)
CRP (mg/L)	7.7 (2–60.4)
Outcome	
Disease severity	44 (30.34)
Death	14 (9.66)

As shown in Table 2, compared with non-severe cases, severe COVID-19 patients were significantly older (median age, 63 vs 40 years; P-value < 0.001), predominantly male (79.55% vs 39.6%; P-value < 0.001), and characterized by a high proportion of comorbidities including hypertension (43.18% vs 16.83%; P-value = 0.001), diabetes (22.73% vs 7.92%; P-value = 0.025), cardiovascular disease (25% vs 4.95%; P-value = 0.001), and other disease (36.36% vs 14.85%; P-value = 0.007). They also presented higher rates of respiratory symptom than non-severe cases (79.55% vs 46.53%; P-value < 0.001). The biological comparison found significant differences for neutrophil count (median, 4.74 vs 3.56; P-value < 0.001), lymphocyte count (median, 1.02 vs 1.73; P-value < 0.001), eosinophil count (median, 0.01 vs 0.04; P-value < 0.001), and CRP level (median, 86.4 vs 3.4; P-value < 0.001).

**Table 2:** Characteristics of the study population according to their disease severity. ENT, ear, nose and throat; CRP, C-reactive protein.

	Non-severe COVID-19N = 101	Severe COVID-19 N = 44	P-value
Demographics			
Age, years, Median (IQR)	40 (26–57)	63 (53.5–72)	0.000
Male, N (%)	40 (39.6)	35 (79.55)	0.000
Comorbidities			
Hypertension, N (%)	17 (16.83)	19 (43.18)	0.001
Diabetes, N (%)	8 (7.92)	10 (22.73)	0.025
Cardiovascular disease, N (%)	5 (4.95)	11 (25.00)	0.001
Respiratory disease, N (%)	7 (6.93)	7 (15.91)	0.125
Other disease, N (%)	15 (14.85)	16 (36.36)	0.007
Clinical symptoms			
Fever, N (%)	38 (37.62)	24 (54.55)	0.069
General symptom, N (%)	35 (34.65)	21 (47.73)	0.144
Respiratory symptom, N (%)	47 (46.53)	35 (79.55)	0.000
ENT symptom, N (%)	32 (31.68)	10 (22.73)	0.323
Digestive symptom, N (%)	22 (21.78)	12 (27.27)	0.525
Blood routine			
Leucocyte (×10^9^ per L)	5.93 (4.63–7.27)	6.62 (5.45–8.15)	0.074
Neutrophil (×10^9^ per L)	3.56 (2.28–4.86)	4.74 (3.4–6.8)	0.000
Lymphocyte (×10^9^ per L)	1.73 (1.3–2.25)	1.02 (0.72–1.36)	0.000
Monocyte (×10^9^ per L)	0.46 (0.38–0.59)	0.49 (0.35–0.7)	0.658
Eosinophil (×10^9^ per L)	0.04 (0.01–0.08)	0.01 (0–0.04)	0.000
Haemoglobin (g/L)	137 (130–149)	141 (123.5–147.5)	0.636
Platelet (×10^9^ per L)	232 (194–280)	210.5 (167–291.5)	0.398
CRP (mg/L)	3.4 (1.08–16.7)	86.4 (21.69–145.8)	0.000
Outcome			
Death	0	14 (31.82)	0.000

**Figure 1:**
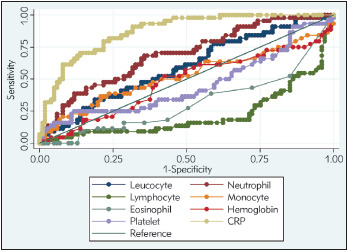
Receiver operating characteristic (ROC) curves of parameters of blood routine for the diagnosis (discriminating) of disease severity on admission. CRP, C-reactive protein.

According to ROC curves of hematologic parameters and CRP admission level, the AUCs of leucocyte count, neutrophil count, monocyte count, hemoglobin count, and CRP level were 0.594, 0.691, 0.523, 0.475, and 0.872, respectively (Figure 1). The AUC for severity prediction of CRP was significantly higher than leucocyte count (P-value < 0.001), and neutrophil count (P-value < 0.001).

As represented in Table 3, CRP level was associated with COVID-19 severity in the univariate analysis (OR=1.22, 95% IC (1.13-1.33)). For the multivariate analysis, we found that CRP level was independently associated with COVID-19severity (OR=1.11, 95% IC (1.01-1.22) for the multivariate regression model; and OR=1.13, 95% IC (1.04-1.23) for the stepwise multivariate regression model).

**Table 3:** Independent discriminators (predictors) of disease severity. OR, odds ratio; CI, confidence interval; ENT, ear, nose and throat; CRP, C-reactive protein. Note: eosinophil count is multiplied by ten, and the CRP level is divided by ten in order to produce ORs that are easier to read.

	Univariate OR (95% CI)	Multivariate Model OR (95% CI)	Multivariate Model (stepwise) OR (95% CI)
Demographics			
Age, years	1.07 (1.04–1.10)	1.04 (1.00–1.08)	1.05 (1.02–1.09)
Male	5.93 (2.58–13.66)	3.90 (1.24–12.33)	3.35 (1.20–9.36)
Comorbidities			
Hypertension	3.76 (1.70–8.29)	0.88 (0.24–3.21)	
Diabetes	3.42 (1.25–9.38)	0.95 (0.21–4.27)	
Cardiovascular disease	6.40 (2.07–19.79)	3.74 (0.76–18.29)	
Respiratory disease	2.54 (0.83–7.74)		
Other disease	3.28 (1.44–7.46)	3.19 (0.96–10.55)	
Clinical symptoms			
Fever	1.99 (0.97–4.08)		
General symptom	1.72 (0.84–3.54)		
Respiratory symptom	4.47 (1.95–10.25)	4.26 (1.31–13.89)	3.11 (1.11–8.74)
ENT symptom	0.63 (0.28–1.44)		
Digestive symptom	1.35 (0.60–3.04)		
Blood routine			
Leucocyte (×10^9^ per L)	1.15 (1.00–1.32)		
Neutrophil (×10^9^ per L)	1.36 (1.14–1.61)	1.01 (0.76–1.34)	
Lymphocyte (×10^9^ per L)	0.33 (0.18–0.60)	0.60 (0.33–1.07)	
Monocyte (×10^9^ per L)	2.11 (0.57–7.88)		
Eosinophil (×10^9^ per L)	0.35 (0.14–0.83)	0.61 (0.22–1.75)	
Haemoglobin (g/L)	0.99 (0.97–1.01)		
Platelet (×10^9^ per L)	1.00 (1.00–1.00)		
CRP (mg/L)	1.22 (1.13–1.33)	1.11 (1.01–1.22)	1.13 (1.04–1.23)

With a cut-off value of 10 mg/L, CRP exhibited sensitivity of 86.36%, specificity of 70.3%, positive predictive value (PPV) of 55.88%, and negative predictive value (NPV) of 92.21%.

## Discussion

Our hospital took care of the first cases of COVID-19 in Casablanca city, Morocco. Since February 2020, 145 cases were admitted including 44 severe cases. Among the severe cases, 14 patients died. The assessment of the disease prognosis and factors of severity are therefore necessary to guide the appropriate therapeutic strategy and reduce the mortality rate especially in a developing country with limited medical resources.

A pattern of hematologic, biochemical, inflammatory, and immune biomarker abnormalities has been identified in patients with severe disease compared to mild systemic disease, and warrant inclusion in risk stratification models. In the present study, based on the analysis obtained from 145 Moroccan patients with COVID-19, we assessed the impact of clinical and biological factors on the severity of the COVID-19 disease [15]. The purpose of this study was to evaluate, according to routine tests usually prescribed on admission, the main parameters that can be used for the rapid assessment of severity.

According to the comparison based on the severity of the disease, our results detected the effect of several reported indicators for disease severity and prognosis. Indeed, we found significant differences image, gender, comorbidities, respiratory symptom, neutrophil count, lymphocyte count, eosinophil count, and CRP level. Our results were similar to what was recently published: clinically severe COVID-19 patients were older and with more comorbidities and breath complications than non-severe patients [16] [17]. In a recent meta-analysis, lymphopenia was defined as a good biomarker associated with an increased risk of severe COVID-19 infection [18]. Regarding our results, higher lymphopenia level was found in the severe cases, but the assessment of the prognosis value of the biological parameters in correlation with the severity of the disease demonstrated that CRP level was more relevant.

The interpretation of ROC curves of hematological parameters and CRP level in our study confirmed that the CRP was a robust predictor of adverse disease outcome. CRP was also an independent discriminator of severe/critical illness on admission in comparison with other biological factors. These results were in agreement with similar report of Luo et al. in Wuhan, which found an AUC of CRP for discriminating disease severity on admission at 0.783. With a cut-off value of 41.3, CRP exhibited similar results of our study with sensitivity of 65%, specificity of 83.7%, positive predictive value (PPV) of 81.6%, and negative predictive value (NPV) of 68.2% [19].

Moreover, some studies evaluated the severity prediction of the COVID-19 using different factors such as age, gender, comorbidities, high neutrophil count, lymphopenia, high CRP level, and high lactate dehydrogenase level. However, the earliness, sensitivity, specificity, and selection of the best independent factor is still under study. None of these factors were considered as an independent and sensitive prognosis factor. Many biomarkers of inflammation and infection were identified as factors of disease severity [8] [20] [7] [21]. The pathological mechanism of COVID-19 is not well elucidated, but the part of inflammation was considered having a primordial role in evolution of the disease [21] [22]. CRP is an acute-phase protein that serves as an early marker of inflammation or infection [23] [24] [25]. Generally, the level is much higher in bacterial infections. The protein is synthesized in the liver and is normally found at concentrations of less than 10 mg/L in the blood [23] [24]. During infectious or inflammatory disease states, CRP levels can activate the classical complement cascade of the immune system and modulates the activity of phagocytic cells, supporting the role of CRP in the opsonization of infectious agents and dead or dying cells [26]. In COVID-19, the exact effect of CRP remains unclear, but it was reported that their level can be used for early diagnosis of pneumonia [10], and assessment of severe pulmonary infectious diseases [27].

Our findings were consistent with recent publications, which indicated that the CRP level on admission was a sensitive and early indicator for COVID-19 severity [19] [28] [29]. Moreover, CRP level was positively correlated with lung lesion at tomographic scans [30]. In common practice for urgent stratification, it is difficult to perform a large panel of biological analyses on admission but the routine test of CRP could predict disease severity and guide management of COVID-19 patients to different extents.

Our study has many strengths. We included all admitted patients for COVID-19 disease. Our data were collected by a trained team of physicians from electronic medical records of patients. Medical investigators collected and reviewed all patient data which made it of high quality. Notably, we had no missing values about the variables included in the analysis. Further, we had access to different patient characteristics including demographics, comorbidities, clinical symptoms, and routine laboratory markers. However, our study may have some limitations. First, it was a retrospective single-centre study. Second, only 145 patients were included and a larger cohort study may verify our results. Third, we did not include other inflammatory biomarkers, such as interleukin 6 or procalcitonin, because they were performed after admission and in few severe cases only.

## Conclusion

Compared to other routine laboratory parameters, we found that CRP level was significantly associated with COVID-19 severity. Therefore, CRP on admission represents a simple and independent factor that can be useful for early detectionofCOVID-19 severity and thereby facilitates the guidance of treatment decisions.

### Author Contributions

CE, MA, SN and FO provided the data. SZ analysed the data. MA and SZ interpreted the data. MA and SZ wrote up the manuscript. CE, SN and FO read and reviewed the final manuscript.

### Data Availability Statement

The authors declare that the data is not publicly available.

### Funding

The authors declare the existence of no support or funding.

## Conflict of interest statement

The authors stated that they have no conflicts of interest regarding the publication of this article.
